# Amyand hernia repair with mesh and appendectomy

**DOI:** 10.1186/s40792-019-0600-2

**Published:** 2019-03-15

**Authors:** Katelin Holmes, Joseph E. Guinn

**Affiliations:** 1 Department of Surgery, University of North Texas Health Science Center, 900 8th Avenue, Fort Worth, TX 76104 USA; 2 Department of Surgery, Medical City Fort Worth, 900 8th Avenue, Fort Worth, TX 76104 USA

**Keywords:** Amyand hernia, Lightweight mesh repair, Incidental appendectomy, Inguinal hernia

## Abstract

**Background:**

Amyand hernias have been described in case reports in the literature and are a rare occurrence in the career of a surgeon. Their management is even less well described and often poses problems in the management of repair due to concerns with contamination associated with appendectomy. Here, we describe a patient with an Amyand hernia seen on CT imaging preoperatively, who underwent appendectomy along with a lightweight mesh plug repair of his hernia.

**Case presentation:**

An 88-year-old male presented with a right groin bulge and underwent preoperative imaging which indicated the presence of his appendix within his hernia. He was taken to the operating room electively where an appendectomy was performed due to significant chronic inflammatory changes. A lightweight mesh plug was used to repair the hernia to prevent recurrence. He did well post operatively without any complications.

**Conclusions:**

A review of the literature supports the use of mesh repair in these of hernias even with appendectomy at the time of the hernia repair. This will guide surgeons who encounter this clinical rarity in their practice.

## Background

Though rare to encounter in a general surgeon’s career, the existence and recommendations for the Amyand hernia date back to the eighteenth century in medical history. Claudius Amyand performed the first appendectomy on an 11-year-old boy on December 6, 1735, where he discovered a perforated appendix in an inguinal hernia sac [1]. Due to his contributions, his namesake commonly refers to any inguinal hernia containing the vermiform appendix. Not only did he perform the first appendectomy, but also documented the first “strange content of a hernia” [1]. Upon review of the literature, the incidence of Amyand hernias is rare and estimated to occur in about 1% of inguinal hernias [2]. Even rarer is the incidence of acute appendicitis associated with an Amyand hernia, which is estimated to be 0.1% of cases [1].

Many questions are posed throughout various case studies in the literature regarding operative management, complications, and post-surgical care, and no clear guidelines widely exist due to the rare nature of the diagnosis. The infrequency of the presentation as well as no clear guidelines for surgical treatment make this diagnosis an intriguing and interesting find in one’s career as well as a subject that warrants further study and review.

## Case presentation

**Fig. 1:**
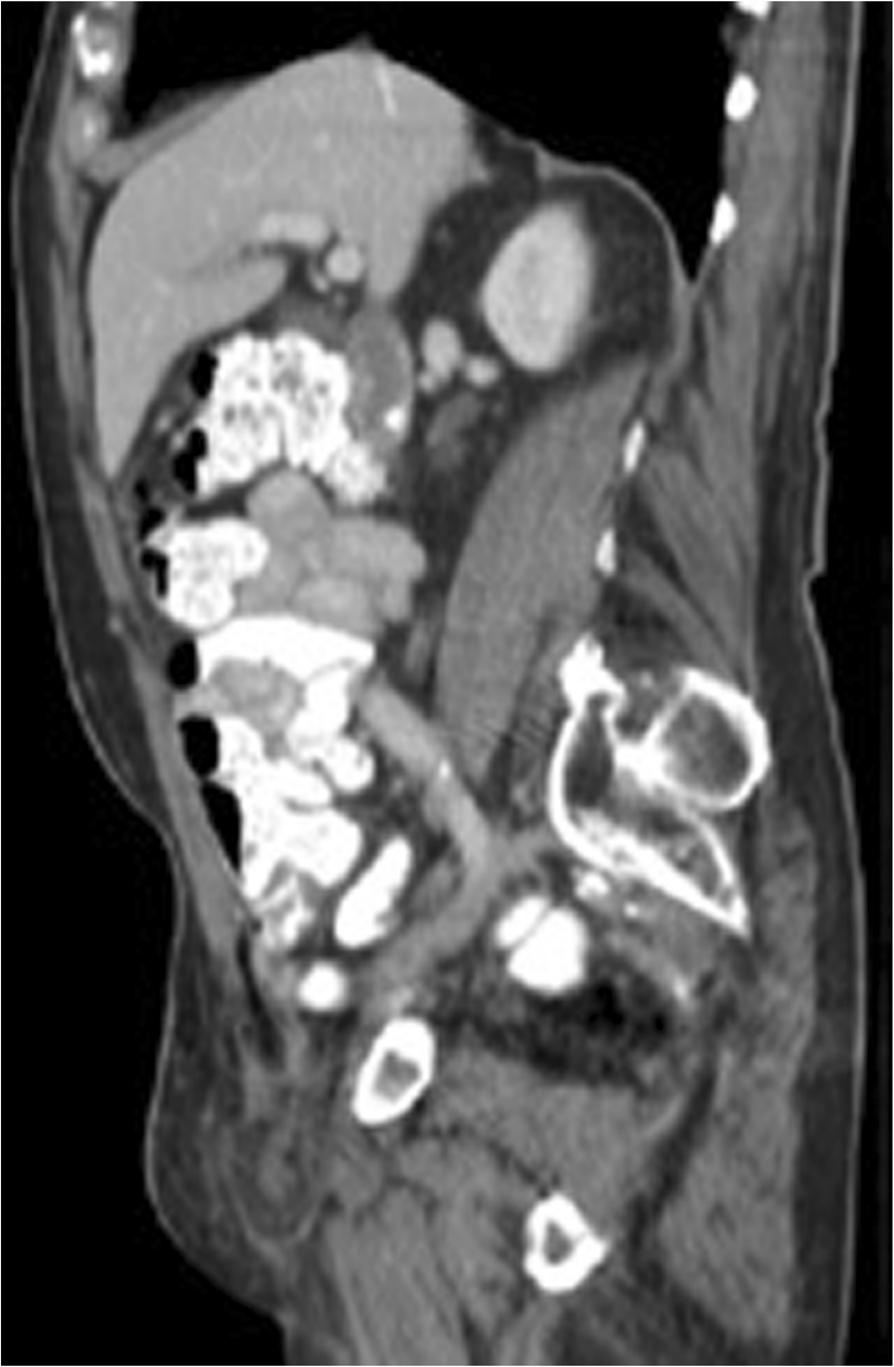
Computed tomography sagittal view of the appendix and omentum within the hernia

**Fig. 2:**
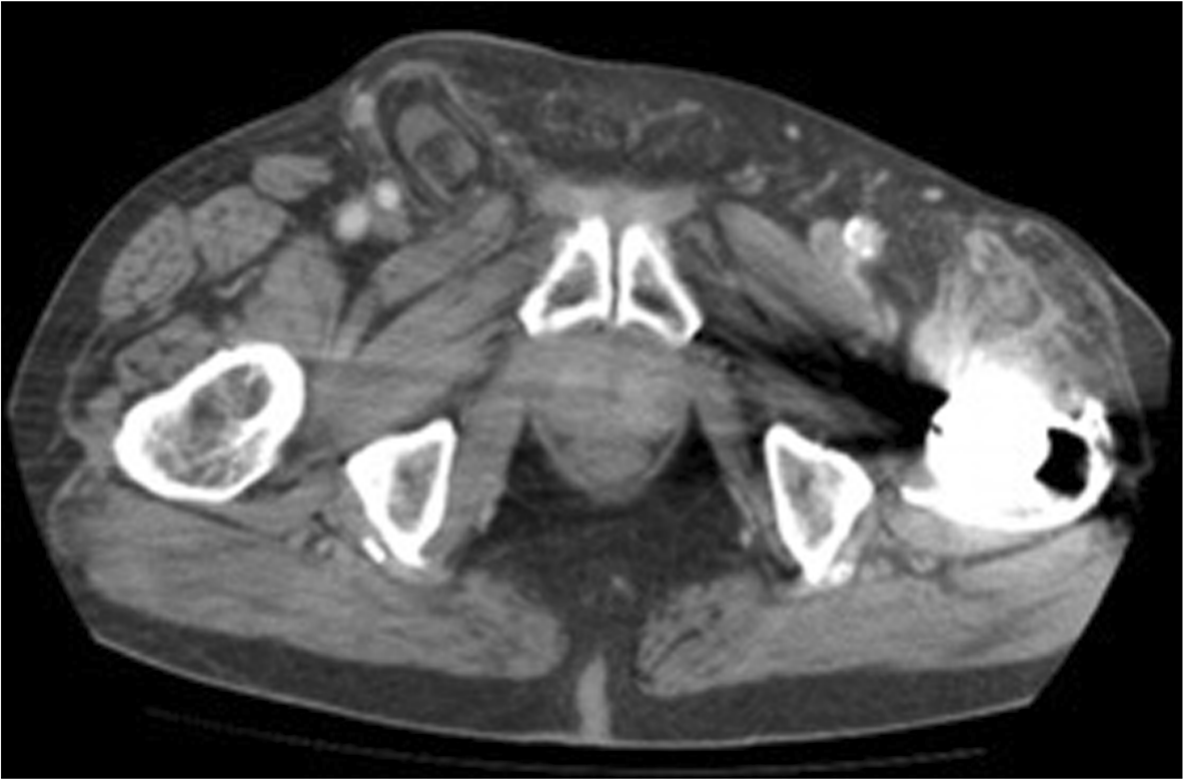
Computed tomography anterior-posterior view of described Amyand hernia

**Fig. 3:**
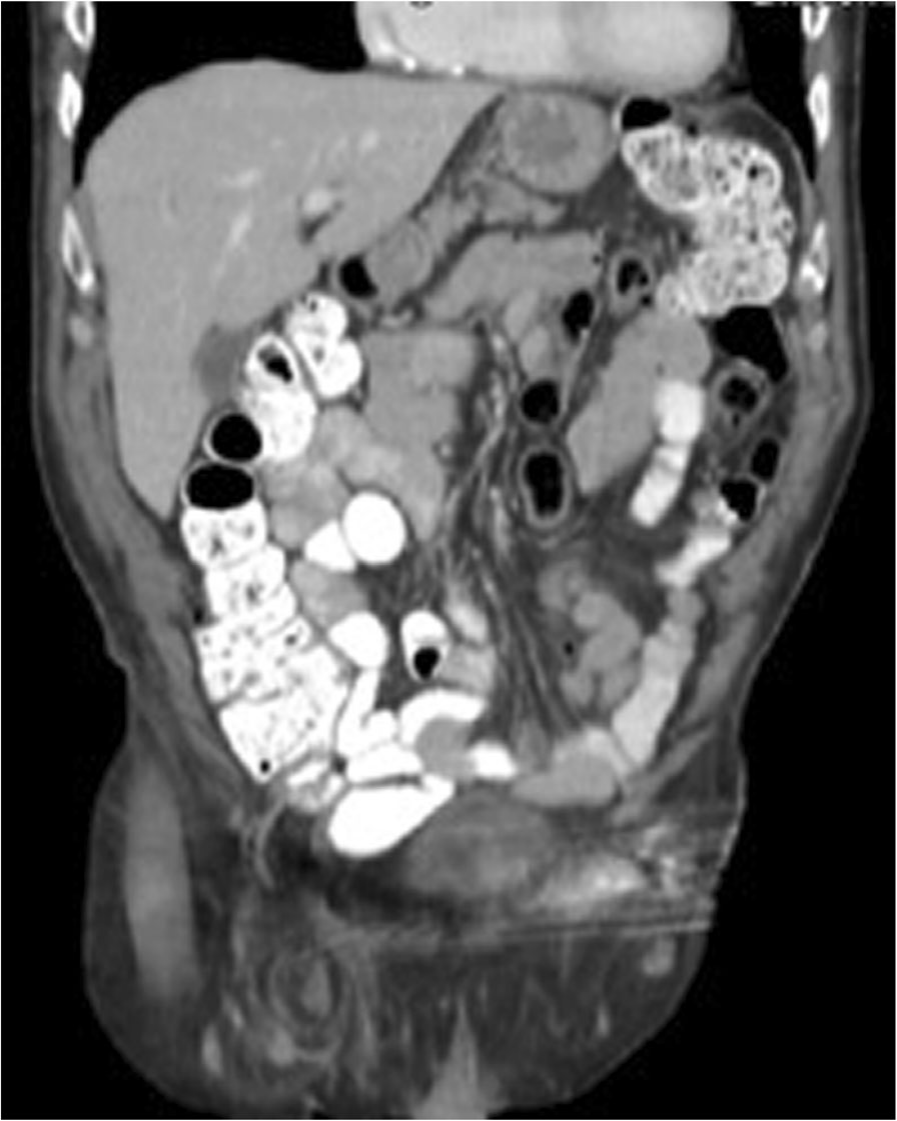
Computed tomography coronal view of described Amyand hernia

An 88-year-old male presented in the outpatient surgical setting with a chief complaint of a right groin bulge that had been present for 6 weeks. He had sharp pain initially when he first developed the abnormality but had been asymptomatic ever since. He did not recall any inciting factors. He was concerned that a previously repaired right inguinal hernia had recurred from its original tissue repair in 1977. Details of the original right inguinal hernia repair were unknown to the patient, other than no implantation of mesh occurred. On physical examination, a 3 cm × 3 cm firm, nontender mass was palpable in the right groin just lateral to the pubic tubercle. A computed tomography scan of the abdomen and pelvis was performed to elucidate the cause of the mass in his groin (Figs. 1, 2, and 3). The imaging was relevant for a right inguinal hernia with the appendix present within the sac. Preoperative laboratory testing revealed a white blood cell count of 4.7 × 10^9^/L. The patient elected to proceed with surgical intervention for hernia repair.

The patient presented to the hospital setting for his elective right inguinal hernia repair. A classic oblique incision was made in the right groin using the anterior superior iliac spine and pubic tubercle as landmarks. The external oblique aponeurosis was opened and the hernia isolated and examined. The hernia was noted to be comprised of an extremely hard and dense amount of omentum that had a chronic, scarred appearance. The base of the appendix could be seen exiting the internal inguinal ring, but the densely adhered omentum prevented reduction of the appendix back into the peritoneal cavity. Initially, there was no indication to perform an appendectomy at the time of the procedure if the appendix could be successfully reduced into the abdominal cavity. However, the chronic appearing adhesions in the area prevented this step. In order to reduce the appendix at that point, a relaxing incision was then made in the typical transverse fashion in the right lower quadrant through the rectus sheath, and the peritoneum entered. The appendix was clearly visualized exiting the abdominal cavity into the inguinal defect. The appendix and its adhered omentum were then carefully reduced back into the abdominal cavity using intraperitoneal countertension without any rupture or spillage. Due to its densely adherent chronic inflammatory tissue, an incidental appendectomy was performed as there was significant tension on the cecum after placing the appendix back in its anatomical location. There was concern for the development of appendicitis post operatively due to the manipulation performed during the procedure. The appendix was then stapled at its base using a standard gastrointestinal anastomosis stapler and passed off the field. The indirect hernia defect was very small and closed with a medium size lightweight mesh plug. The patient was discharged from the post-anesthesia care unit the same day as surgery and had no complications from his recovery course. No additional antibiotics were given other than a single prophylactic dose during the surgical case. At his 2-week follow-up, he had no recurrence of his hernia and was doing well. On pathologic examination, there was no evidence of appendiceal inflammation or appendicitis. The periappendiceal fat did exhibit some fat necrosis, however, supporting the chronic periappendiceal adhesive changes.

## Discussion

Due to the rarity of Amyand hernias, the surgical management may pose somewhat of a challenge for general surgeons as the majority of these are discovered at the time of operation. If acute appendicitis is present in conjunction with an Amyand’s hernia, the classical recommendation has been to proceed with appendectomy and herniorrhaphy, along with mitigating the risk of sepsis by using antibiotics, irrigation, and possible drainage [3]. As high as a 50% infection rate may exist following their repair when the appendix is acutely inflamed [3], making repair with mesh controversial. Though a large amount of newer literature exists in the field of surgery advocating the use of mesh in clean-contaminated or contaminated ventral hernia cases, there is a paucity of literature regarding Amyand hernia repair success rates or mesh use. As such, though possible to extrapolate recommendations from other fields of study, this must be cautiously considered. A large part of the surgical literature regarding Amyand hernias predates this newer mesh debate, and as such, many of the described repairs are tissue based in nature.

Another decision that must be considered is whether to proceed with incidental appendectomy when the intraoperative diagnosis is made and a patient has a normal appearing appendix. The benefits to proceeding with appendectomy can avoid additional future operations with their respective morbidities [1]. However, the concern for introducing contamination into the case is problematic.

In 2007, Losanoff and Basson proposed an Amyand hernia classification system with recommendations for surgical management that may prove very useful for intraoperative decision making for such a diagnosis [4] (Table 1). Certainly, these must be guided with the hallmark principles of general surgery—critical thinking and common sense—and adapted on a case by case basis. By applying knowledge of lightweight meshes in the literature with clean-contaminated cases, one can consider performing mesh repair as a modern surgical management technique as opposed to the older case reports that support tissue repair. According to this publication, when a normal appendix is encountered and does not exhibit inflammatory changes (type 1), an appendectomy versus reduction may be performed and mesh may be used in the hernia repair. However, while our patient’s appendix did not demonstrate an appearance of appendicitis, it did exhibit periappendiceal adhesions in the hernia sac, which not only complicated its reduction, but also did not place this patient’s presentation into the classification system described by Losanoff and Basson.

**Table 1:** Types of Amyand hernias and their management [4]

Classification	Description	Surgical management
Type 1	Normal appendix within an inguinal hernia	Hernia reduction, mesh repairs, appendectomy in young patients
Type 2	Acute appendicitis within hernia, no abdominal sepsis	Appendectomy through hernia primary repair of Hernia, no mesh
Type 3	Acute appendicitis within an inguinal hernia, abdominal wall, or peritoneal sepsis	Laparotomy, appendectomy, primary repair of hernia, no mesh
Type 4	Acute appendicitis within an inguinal hernia, related or unrelated abdominal pathology	Manage as type 1 to 3 hernia investigate or treat second condition as appropriate

A recent publication by Kose et al. retrospectively examined a five-patient case series of Amyand hernias with appendectomy for a normal appearing appendix and an inguinal hernia repair with mesh placement [5]. In this series, there were no post-operative complications or hernia recurrence within a year. The authors of this series also discuss the management of the appendix whose fibrous connections within the hernia sac prove difficulty in dissection and agree with appendectomy at the time of the index operation. Their description of “secondary appendicitis” after manipulation mimics the presentation of our patient whose fibrous adhesions prevented simple reduction without more significant dissection. This further supports the management our report describes as the same presentation was encountered with our patient.

The diagnosis of an Amyand hernia, though many general surgeons may never encounter in their careers, proves to be an unusual challenge for those that come across it. Ranging from the pediatric population to the elderly, this is a differential diagnosis that albeit rare should remain on one’s radar when encountering presentations that fit its description. While historically, the management and repair of Amyand hernias do not support appendectomy in a normal appearing appendix or implementation of mesh in the hernia repair, more modern literature certainly creates a basis for this type of surgical management. Critical thinking, patient adaptation, and common sense will continue to guide the decision management for a large majority. The detailed information that various case reports and literature reviews provide are invaluable to those of us who find ourselves in these situations and will guide us and prepare us for the day that we are entrusted with the care of a patient with this type of challenging case.

## Conclusions

Some Amyand hernias may be managed with mesh repair of the hernia while performing appendectomy at the time of the index operation. Newer surgical literature supports such decision-making with positive patient outcomes.
